# The Crossroad of Ion Channels and Calmodulin in Disease

**DOI:** 10.3390/ijms20020400

**Published:** 2019-01-18

**Authors:** Janire Urrutia, Alejandra Aguado, Arantza Muguruza-Montero, Eider Núñez, Covadonga Malo, Oscar Casis, Alvaro Villarroel

**Affiliations:** 1 Biofisika Institute (CSIC, UPV/EHU), University of the Basque Country, 48940 Leioa, Spain; janire.urrutia@gmail.com (J.U.); alejandra.aguado@ehu.eus (A.A.); arantza.muguruza.montero@gmail.com (A.M.-M.); enviadero@gmail.com (E.N.); covadonga.m@gmail.com (C.M.); 2 Departamento de Fisiología, Facultad de Farmacia, Universidad del País Vasco (UPV/EHU), 01006 Vitoria-Gasteiz, Spain; oscar.casis@ehu.eus

**Keywords:** calmodulin, ion channels, channelopathies, calcium

## Abstract

Calmodulin (CaM) is the principal Ca^2+^ sensor in eukaryotic cells, orchestrating the activity of hundreds of proteins. Disease causing mutations at any of the three genes that encode identical CaM proteins lead to major cardiac dysfunction, revealing the importance in the regulation of excitability. In turn, some mutations at the CaM binding site of ion channels cause similar diseases. Here we provide a summary of the two sides of the partnership between CaM and ion channels, describing the diversity of consequences of mutations at the complementary CaM binding domains.

## 1. Universal Calcium Signaling

Calcium is a universal signaling messenger involved in fundamental processes, including muscle contraction, long-term potentiation, apoptosis, or cell proliferation [1,2]. While Ca^2+^ concentration at the extracellular milieu or at intracellular storage compartments is of the order of 2 mM, intracellular Ca^2+^ is kept in the low-to-mid nanomolar range, establishing a step 20,000-fold gradient. Since Ca^2+^ is toxic to the cell, signaling by this cation has to be brief and tightly controlled [2]. Upon cellular stimulation, different mechanisms lead to transient intracellular increases in Ca^2+^ concentrations that initiate different protein activities. Most proteins are devoid of Ca^2+^ binding sites, requiring a mediator to respond to this cation [1]. The ability to transmit conformational changes to a large and diverse array of proteins in response to Ca^2+^ oscillations, coordinating the activity of hundreds of proteins, makes calmodulin (CaM, see a list of Abbreviations) the most important Ca^2+^ signal transducer in eukaryotic cells. CaM, initially named, among others, Ca^2+^-dependent regulator (CDR), was discovered in 1970 by Cheung and Kakiuchi as a Ca^2+^-dependent regulator of cyclic nucleotide phosphodiesterase in the brain [3,4,5]. Since then, the number of proteins and functions regulated by CaM keeps on expanding, including kinases, phosphatases, metabolic enzymes, ion channels, pumps, transcription factors, and many other proteins from yeast to humans [6]. The ability to bind and regulate such a large and diverse array of proteins resides in its inherent flexible nature that allows to fit structurally into more than 300 target proteins and to trigger diverse regulatory mechanisms.

## 2. Calmodulin Links Chemical and Electrical Signals

The linkage between Ca^2+^, CaM, and cellular electrical properties was revealed soon after CaM discovery through the genetic study of the avoidance response in *Paramecium tetraurelia* [7]. *Paramecium* is a unicellular eukaryotic organism uniformly covered with simple cilia that propels the cell in a spiral movement. When an obstacle is encountered or a stimulus is received, *Paramecium* swims backwards for about one second, before resuming its forward progress. In the 1970–90s of the past century, genetic variants that differed in their “avoiding reaction” were isolated, some underreacting and others overreacting [7]. Functional analysis underscored that the basis of such contrasting responses were deficiencies on a Na^+^ inward current in the first case, or a deficit in a K^+^ outward current for the overreacting variants. Interestingly, both currents are Ca^2+^-dependent and genetic analysis demonstrated that defects on the sole CaM gene in *Paramecium* were responsible for both phenotypes [8]. Thus, mutations in the same CaM gene result in opposing behavioral responses. How is that possible?

**Figure 1:**
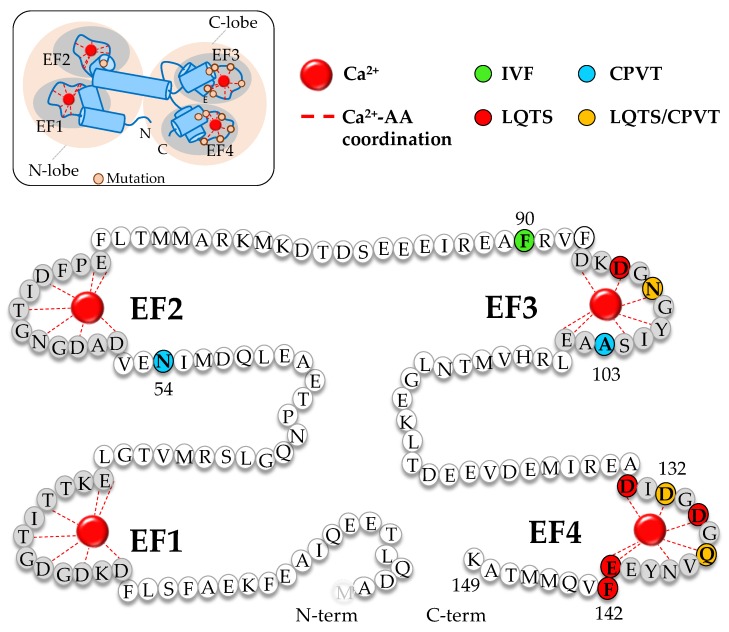
Location of the different mutations in calmodulinopathies. Top left panel: schematic representation of the structure of CaM showing Ca^2+^ coordination and mutations found in calmodulinopathies. The grey areas highlight the EF-hands shown in the main panel. Disease-associated residues are colored in green (IVF: idiopathic ventricular fibrillation), blue (CPVT: catecholaminergic polymorphic ventricular tachycardia), red (LQTS: long QT syndrome) and orange (both: LQTS/BrS) in the CaM sequence. The Ca^2+^-binding loop of each EF-hand is emphasized with a grey background.

CaM is one of the most conserved proteins in evolution, with a sequence of 148 amino acids that is identical in all vertebrates (the first methionine is lost in the mature protein). It is encoded by three independent genes (CALM1-3) in humans, all translating identical CaM sequences. It is composed of four Ca^2+^-binding EF-hands, and each contains a helix-loop-helix motif with a central acidic loop of 12 residues for Ca^2+^ coordination via at least six oxygen atoms (Figure 1). The EF-hands are structurally organized in globular pairs forming the N- and C- terminal lobes that fold independently. Although both lobes probably appeared as a result of a gene duplication and are very similar in sequence and structure, the affinity of the C-terminal lobe is one order of magnitude higher than that of the N-terminal lobe [9]. The EF-hands can adopt any of three conformations, open, semi-open and closed. The angles between the lobes and within the helix–loop–helix EF-hand open upon Ca^2+^ binding, exposing patches of hydrophobic amino acids. Whereas the interhand angle differs when loaded with Ca^2+^ in the C-lobe, such a conformational change is not observed for the N-lobe [10]. Both apo- (Ca^2+^-free) and holo-CaM (Ca^2+^-loaded) can bind and regulate target proteins [11]. The CaM-binding domains (CaMBD) are usually amphipathic helical structures rich in hydrophobic and basic residues. The flexibility of the abundant methionine side chains confers another level of plasticity, enabling CaM to adapt to many target molecules and to display diverse regulation mechanisms in response to different Ca^2+^ levels [12]. CaM conformation also changes in the presence of target proteins, resulting in a sophisticated interplay between Ca^2+^ and target affinities: Ca^2+^ affinity of each lobe increases or decreases when CaM engages with a target, and the affinity for some targets increases when loaded with Ca^2+^, but for others, it decreases. For instance, the affinity of the N-lobe for Ca^2+^ is higher than that of the C-lobe when engaged with voltage-gated potassium channels type 7 (K_V_7), whereas Ca^2+^ does not bind to the C-lobe when CaM is engaged with some small conductance calcium-activated potassium (SK) channel variants. In other words, the N-lobe becomes insensitive to Ca^2+^ oscillations when coupled to K_V_7 channels, whereas the C-lobe becomes indifferent to Ca^2+^ when coupled to SK2 channels. When the C-lobe is loaded with Ca^2+^, the strength of CaM binding to K_V_7 channels decrease, whereas when the N-lobe is loaded with Ca^2+^, the affinity for SK channels increases [13,14,15,16,17]. Our understanding of CaM function is limited, and anticipating the magnitude and direction of the changes on Ca^2+^ affinities prompted by the target and mutations remains a challenge, even knowing the structure of the complex at atomic resolution. As a general rule, the C-lobe plays a predominant role in target recognition under resting low Ca^2+^ conditions, and often the interaction between the target and the C-lobe weakens, whereas the binding to the N-lobe becomes stronger when each lobe becomes loaded with Ca^2+^ [10].

The *Paramecium* genetic variants revealed a functional bipartition in which the N- and C-lobes have separate missions [18]. Mutations in the C-lobe impaired the Ca^2+^-dependent activation of a K^+^ outward current, whereas mutations located in the N-lobe disrupted a Na^+^ inward current. These studies underscored the importance of CaM in membrane electrogenesis. The possibility that alterations on the activity of ion channels due to mutations in different lobes of CaM could alter human physiology was corroborated in 2012 [19], which is remarkable given that defects in just one of the six alleles can cause disease. The descriptions of the pathological consequences of mutations in CALM genes have focused on its cardiac manifestations [20] (Figure 1).

## 3. Cardiac Calmodulinopathies

Sudden cardiac death (SCD) is the main cause of death in the western countries [21]. Multiple and collaborative causes—such as myocardial infarction, coronary ischemia, endocrine diseases—contribute to SCD in adults. All these alterations increase the susceptibility to lethal ventricular arrhythmias. However, in young people (infants, children, and adolescents) the cause of SCD is more often genetic. Many gene alterations that predispose to ventricular lethal arrhythmias have been described, which include mutations in the main cardiac Na^+^, Ca^2+^, and K^+^ channels [22], and mutations in other regulatory proteins such as ankyrin [23], caveolin [24], AKAP [25] and, more recently, CaM [19,26].

CaM regulates almost every cardiac ion channel, either by direct protein-protein interactions or indirectly through CaM-kinase II [27,28]. Thus, any alteration in CaM expression or function can derange the electrical behavior of the heart and induce arrhythmia or SCD [29,30]. The most recently described arrhythmic syndromes associated to mutations in CaM have been named “Calmodulinopathies” [31,32,33]. Pathological variants on every CALM gene have been discovered, and some have been isolated from different genes (e.g., CALM1-D130G, CALM2-D130G and CALM3-D130G; CALM1-N98S and CALM2-N98S, or CALM1-F142L and CALM3-F142L [34,35]). Nearly all of the identified CaM mutations impair Ca^2+^ binding, but little is known on how they affect the physical interaction with many relevant cardiac or neuronal ion channels (see below). Except one (N54I), all other seventeen amino acid replacements so far described are located in the C-lobe and connected with different cardiac dysfunctions, some associated with sudden cardiac arrest following exercise or emotion [35] (Figure 1). Some mutations have been structurally analyzed, and found to cause variable conformational changes of the whole C-lobe [36]. Mutations in CaM can be lethal at very early ages, and this can be a possible reason for the low prevalence of these calmodulinopathies. In fact, only 30 patients have been described carrying CALM mutations, where 20 were de novo mutations and only three inherited (N54I, F90L and A103V) [32]. The redundant nature of the CALM genes implies very strong pressure to conserve the identity of the two lobes. Perhaps most mutations in the N-lobe are not compatible with human life, explaining that only one pathological variant has been detected in this lobe until now. Interestingly, the only mutation found in the N-lobe, N54I, has relatively minor effects on CaM properties. The clinical phenotypes include Long QT Syndrome (LQTS), Catecholaminergic polymorphic ventricular tachycardia (CPVT), and idiopathic ventricular fibrillation (IVF) [20,33].

Excitation–contraction coupling (ECC) in cardiac muscle is initiated by activation of l-type voltage-gated Ca^2+^ channels (Ca_V_). Ca^2+^ entry through these channels causes the opening of cardiac ryanodine receptor ion channels (RyR2) promoting a massive Ca^2+^ release (Ca^2+^-activated Ca^2+^-release) from the sarcoplasmic reticulum (SR) that initiates muscle contraction. The subsequent Ca^2+^ sequestration back into the SR by the ATPase-associated type-2a pump SERCA2a leads to muscle relaxation. Na^+^ entry through Na^+^ channels results in electrical excitation, thereby initiating ECC. The Na^+^-Ca^2+^ exchanger (NCX), on the other hand, electrogenically exchanges 1 Ca^2+^ ion for 3 Na^+^ ions, creating a direct link between Na^+^ influx and Ca^2+^ cycling [37]. Generally, dysregulation of Ca^2+^ handling in the SR underlies CPVT, whereas LQTS involves malfunction of ion channels at the sarcolemma. The activity of all components implicated in the maintenance of Ca^2+^ homeostasis must be fine-tuned and orchestrated for appropriate heart contraction and rhythm.

The main repolarizing currents in the human heart are the transient outward (I_to_), the rapid delayed rectifier (I_Kr_) and the slow delayed rectifier (I_Ks_) K^+^ currents, carried through the K_V_4.3, K_V_11.1 or hERG and K_V_7.1 channels, respectively [22,38]. All these channels have been proposed to be modulated in some way by CaM. However, the hERG channel sequence has no known putative CaM binding motifs and the activation of either CaM or CaMKII have no effects on I_Kr_ amplitude or kinetics [39,40]. Besides, the reported effects of CaM on the I_to_ amplitude or kinetics are mediated by changes in the activity of Ca^2+^-CaM-dependent enzymes, including Ca^2+^/CaMKII [28,41]. In conclusion, the only cardiac voltage-gated K^+^ channels directly modulated by CaM seem to be K_V_7.1 channels. This modulation will be more extensively described in Section 7.

## 4. Cardiac Ryanodine Receptors

CPVT is most commonly caused by gain-of-function RyR2 mutations with excessive Ca^2+^ release [42,43] or mutations affecting RyR2-binding proteins [19,44], which lead to spontaneous opening and Ca^2+^ waves that trigger membrane depolarization. CPVT is an inherited disorder characterized by episodic syncope and/or sudden cardiac arrest during exercise or acute emotion in individuals without structural cardiac abnormalities that is suspected to cause a substantial number of SDCs in young individuals. RyR2 channels are equipped with two built-in Ca^2+^-binding EF-hands. The EF-hands of RyR respond to Ca^2+^ and mediate the gating of its Ca^2+^ channel. Thus, the presence of an external Ca^2+^ sensor is somehow redundant, and CaM plays a modulatory role [45]. CaM has a differential inhibitory effect on RyR2 channel activity at low and high free Ca^2+^ concentration [33]. In a CPVT RyR2 mutant mouse model carrying a point mutation (R2474S), the interaction with CaM upon PKA stimulation is altered [46]. Since CaM binds and regulates RyR2, it has been assumed that is the disruption of this particular interaction which leads to CPVT for the relevant CALM mutants. However, studies in mice models are difficult to reconcile with this view (see below).

RyR2 channels are huge homotetrameric structures exceeding 2 million Daltons in molecular weight composed of many regulatory sub-domains. Mutagenesis analysis indicates that both apo-CaM and holo-CaM binds to a site comprised by residues 3580-3609 of mouse RyR2. Most of the mutations responsible for different cardiac diseases are clustered in the N-terminus, central and C-terminal regions of the RyR2 primary sequence, and no mutations in the CaMBD linked to disease have yet been reported [43]. Two mice models that disrupt the CaMBD can bring some light on this issue. A knock-in carrying a triple mutation (W3587A, L3591D, and F3603A) interrupts direct CaM binding [47], but, surprisingly there are no major cardiac disturbances in heterozygosis, and no clear signs of CPVT even in homozygosis. Instead, the animals suffer cardiac hypertrophy and early death in homozygosis. A more focused model carrying single RyR2 amino acid substitution (L3591D) perturbs the CaM interaction at low Ca^2+^ levels, but little functional effect at higher systolic concentrations has been noted. Relatively minor cardiac disturbances have been described for these mice [48]. Thus, perturbations on the RyR2 CaMBD do not recapitulate the consequences of CPVT-causing CALM mutations. Alternatively, the product of those CALM genes may alter RyR2 function indirectly through other targets, including voltage-dependent K^+^, Na^+^, and Ca^2+^ ion channels.

## 5. Cardiac Voltage-Gated Calcium Channels

The arrangement of proteins within biological systems into macromolecular complexes and nanodomains is critical for function. RyR2 channels are organized in the cardiac dyad, a specialized signaling hub responsible of cardiac contraction. Classically, it consists of groups of l-type Ca_V_1.2 channels located in the transverse tubules closely apposed (10–12 nm) across the dyadic cleft to clusters of RyR2 channels on the SR membrane [49]. It is known that proteins in close proximity influence each other’s function. CaM favors the formation of Ca_V_1.2 clusters that cause concerted activation, and this cooperative gating generates zones of high Ca^2+^ influx that amplify the Ca^2+^ signal, an amplification that appears to play a fundamental role in cardiac ECC [50]. These channels are regulated by Ca^2+^-dependent inactivation (CDI), an important feedback system required for Ca^2+^ homeostasis [33]. For both processes, CDI and cooperative gating, the CaM C-lobe plays a crucial role. Indeed, CDI reduction can lead to LQTS [51]. Remarkably, many pathological CALM mutations cause LQTS, and about half of those are reported to manifest also CPVT [20]. LQTS is characterized by a prolongation and/or distortion of the cardiac action potential, and thus the QT interval and T-wave, resulting in a prolonged repolarization. Mutations in any of 17 genes can cause LQTS, which include, among others, voltage-dependent K^+^, Na^+^, and Ca^2+^ channels, and ion channel accessory subunits [52].

A study using human induced pluripotent stem cell-derived cardiomyocytes (hiPSC-CM) from a symptomatic carrier of the CALM1-F142L mutation revealed alterations of CDI and a reduction in the persistent Na^+^ current, with unremarkable alterations on CaM-regulated voltage-dependent K^+^ currents [53]. Other LQTS-causing mutations (D96V, D130G, and F142L) have been found to disrupt CDI in heterologous expression systems, whereas CaM mutants related to CPVT (N54I and N98S) exhibited little or no effect on CDI [51]. The N98S CaM mutant is capable of causing either CPVT [19], LQTS [54] or both [36], suggesting a critical interplay between the action on multiple targets and, perhaps, differing CaM expression levels among patients [51]. There is a marked increase in affinity between CaM and the CaMBD of Ca_V_1.2 channels for the F142L mutant under low Ca^2+^ conditions, consistent with the dominant effect of this mutation [36,51]. However, such an increase in binding affinity is not observed for other mutants. In fact, when mutant and wild type CaM are expressed in ratios that approach genetic balance, which should reduce the fraction of channels with pre-associated CaM variants, little or no effect on CDI is revealed for some mutants [51]. Thus, although the correlation between CDI and LQTS is remarkable, additional mechanisms should also be considered, such as the impact on channel clustering and in other targets, as well as integration of the signal at tissue and organism level.

**Figure 2:**
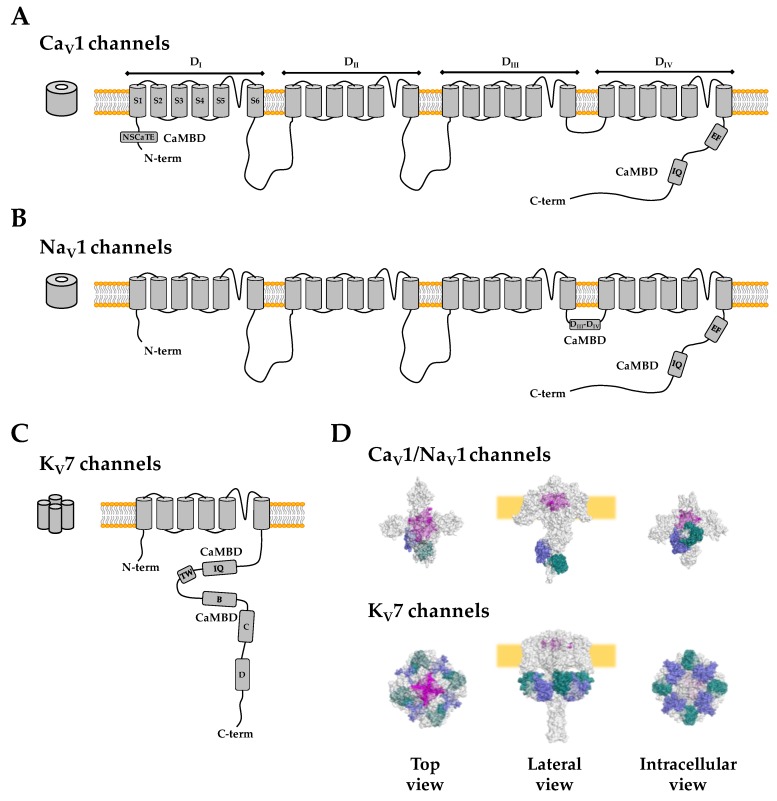
Topology of Ca_V_1, Na_V_1, and K_V_7 channels. Schematic representation of Ca_V_1 (**A**), Na_V_1 (**B**), and K_V_7 (**C**) channels. The functional assemblies are schematically represented on the left side. Note that Ca_V_1 and Na_V_1 channels are composed of four repeats within a single polypeptide, while four subunits are needed to form K_V_7 channels. Homologous repeat domains (D_I_–D_IV_); transmembrane segments (S1–S6); N-terminal spatial Ca^2+^ transforming element (NSCaTE), CaM Binding Domain (CaMBD), IQ motif, EF-hands, TW helix, helix B, helix C, and helix D are labeled. (**D**) Composite 3D structures of Ca_V_1/Na_V_1 channels (top panel; combining PDBs: 5GJW and 4DCK) and K_V_7 channels (bottom panel; PDBs: 5VMS and 3BJ4). The pore forming domain is colored in magenta, the CaM N-lobe in blue and C-lobe in green. Transparency was set to 40%, allowing the view of CaM from the top, and the pore from a lateral perspective. Note that in the presence of Ca^2+^, the N-lobe of CaM should be near the N-terminal of the Ca_V_ channels or the D_III_–D_IV_ linker for Na_V_ channels after activation (not shown). The auxiliary β subunits are not shown. Pymol 1.5 was used to render panel D.

**Figure 3:**
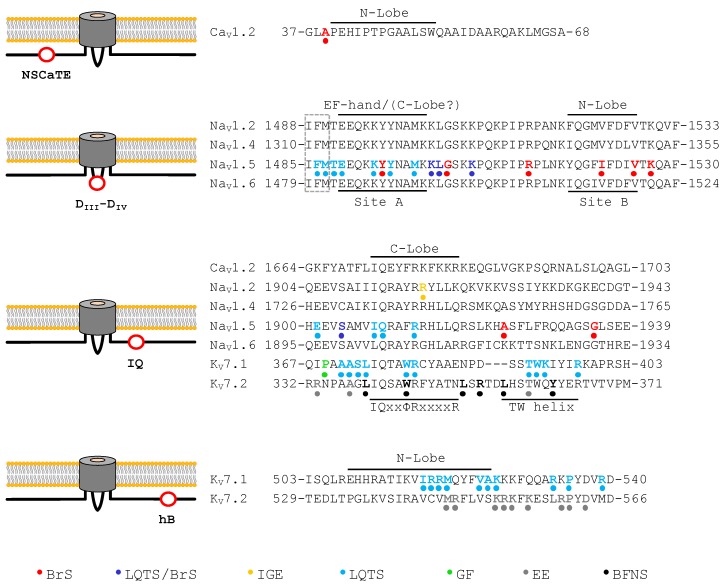
Location of the different mutations found in Ca_V_1, Na_V_1, and K_V_7 channel CaMBD that lead to channelopathies. Left: schematic overall location of different CaMBD (red circle). Note that K_V_7 channels have four IQ/helix A and helix B domains (See Figure 2). NSCaTE (N-terminal spatial Ca^2+^ transforming element) is only present in Ca_V_1 channels, D_III_–D_IV_ repeat domain linker CaMBD is only found in Na_V_1 channels, the IQ motif (IQ) is present in all of them and helix B (hB) is only present in K_V_7 channels. Right: The IFM inactivation motif of Na_V_ is boxed. The interacting CaM lobe or channel domain is indicated on top of the corresponding sequences. The location of the mutation and the disease linked to each mutation is indicated in the color scheme at the bottom. BrS: Brugada syndrome; LQTS: long QT syndrome; IGE: idiopathic generalized epilepsy; GF: gingival fibromatosis; EE: epileptic encephalopathy; BFNS: benign familial neonatal seizures.

The cardiac Ca^2+^ channel macromolecular complex is composed by four subunits arranged in a 1:1:1:1 stoichiometry: α1 (170 kDa), α2 (150 kDa), δ (17–25 kDa), and β (52 kDa). The primary structure of the pore-forming α1 subunit, encoded by the CACNA1C gene, is composed of four homologous repeats (D_I_–D_IV_), each of which consists of six transmembrane segments (S1–S6) linked by variable cytoplasmic loops (Figure 2). CDI is intimately related to a single CaM molecule tethered to the central C-terminal IQ site [55]. CDI requires CaM pre-association to the channel under low resting Ca^2+^ conditions. A conformational change upon Ca^2+^ binding is transmitted to the channel to impose inactivation. It has been proposed that this pre-association of the C-lobe of apo-CaM is required to amplify the action of a limited fraction of pathological CaM molecules, and, thus, molecules like calcineurin and CaM-kinase II may play little role in LQTS [51]. The IQ motif is a ∼11 amino acids long sequence (IQxxΦRxxxxR, where Φ is a bulky hydrophobic residue, see Figure 3) that represents perhaps the most recognizable apo-CaM binding site that interacts with the C-lobe. Upon loading the C-lobe with Ca^2+^, the interaction with the IQ motif generally becomes weaker. The term ‘IQ’ refers to the first two amino acids: isoleucine (commonly) and glutamine (almost invariably) [56]. Most other, but not all, CaM binding sequences can be discovered with the use of knowledge-based methods with a reasonable rate of success [57,58,59]. However, in spite of the large number of complexes resolved at atomic resolution, the orientation of the target, the changes in Ca^2+^ binding affinity and the conformational response is not yet predictable from the sequence alone, not even for the IQ motif [60].

l-type Ca_V_ channels have an N-terminal and a C-terminal CaMBD (Figure 2). A component of CDI depends on the interaction of the Ca^2+^-loaded N-lobe with the N-terminal spatial Ca^2+^ transforming element (NSCaTE) region located in the N-terminal domain between residues 47 and 68 of the channel [61]. A mutation linked to Brugada Syndrome (BrS) located in the N-terminal region (A39V), upstream of the NSCaTE domain, may interfere with N-lobe-dependent CDI [62]. BrS is an inherited cardiac arrhythmic syndrome associated with high risk of ventricular fibrillation and SDC in the absence of structural abnormalities [63]. Alternatively, the pathological manifestation of the A39V variant may be a consequence of general misfolding, since the mutated channels are retained at the endoplasmic reticulum, with gross changes in cellular morphology and a dramatic reduction in current density in CHO-K1 cells [64].

## 6. Cardiac Voltage-Gated Sodium Channels

Whereas no channelopathies within the IQ motif of Ca^2+^ channels have been described, mutations at the equivalent site of voltage-gated sodium channels (Na_V_) have been linked to several excitability disorders [65,66] (Table 1; Figure 3). Both Na_V_ and Ca_V_ channels present a similar architecture (Figure 2), which consists of a 24-transmembrane-α-subunit (4 repeats domains), and differs in the auxiliary β subunit (Na_V_ β1–4). These channels present an IQ CaMBD at the C-terminal domain preceded by a vestigial EF-hand-like domain [67]. Mutations in the IQ motif of Na_v_1.5 (R1897W, E1901Q, S1904L, Q1909R, R1913H, and A1924T) [68,69] result in BrS1, LQT3 or mixed syndromes (an overlap of BrS1/LQT3 [65,70,71], see Figure 3). Besides the IQ site, CaM binds in solution to two sites within a fragment with the sequence of the intracellular loop between repeats 3 (D_III_) and 4 (D_IV_) of Na^+^ channels (Figure 2), an interaction that is postulated to be critical to modulate inactivation mediated by the IFM motif located at the very beginning of the D_III_–D_IV_ linker [72]. It has been suggested that, upon Ca^2+^ elevation, the IQ-bound CaM molecule is transferred to the D_III_–D_IV_ linker or bridge both sites after a 180° re-orientation of the C-lobe around the IQ helix, so the N-lobe could reach alternate sites [60].

Recent cryo-EM single particle reconstitutions reveal that the vestigial EF-hand motif located after the last S6 transmembrane segment, just upstream of the IQ site, interacts with site A of the D_III_–D_IV_ linker [79,80]. In solution, the holo-C-lobe can bind to site B [72], and Ca^2+^-loaded CaM can interfere with the binding between the vestigial EF-hand and the D_III_–D_IV_ linker. As pointed out by Van Petegem and collaborators, whether holo-CaM can modulate this interaction in the full channel remains to be found [77].

Mutations within the D_III_–D_IV_ linker have also been associated with the genetic arrhythmia syndromes BrS and LQTS [74] (Table 1; Figure 3). The consequent reduction in CaM binding results in increased persistent Na^+^ current or slower inactivation [66,81]. However, not every disease-causing mutation in the D_III_–D_IV_ linker leads to a reduction on CaM binding [72]. Another cluster of mutations linked to BrS and LQT is located in the EF-hand like domain (D1790G, Y1795InsD, L1825P, and R1826H) [36,77] (Table 1; Figure 3).

Somehow resembling the role of CaM in cluster formation for Ca^2+^ channels, it has been suggested that CaM may favor the formation of Na_V_ dimers [66,82]. The existence of such Na^+^ channel dimers has received strong experimental support, although the C-terminal region that harbors the CaMBD is not required for dimerization. The protein 14-3-3, instead of CaM, appears to play a critical role for functionally coupling adjacent Na^+^ channels [83].

## 7. Cardiac Voltage-Gated K_V_7.1 Potassium Channels

K_V_7.1 is another CaM binding channel involved in LQTS (Table 2). K_V_7.1 in association with the auxiliary β subunit minK (currently referred to as KCNE1) represents the molecular substrate for the slow delayed rectifier potassium current (I_Ks_) [22]. This current plays a critical role in repolarization of the cardiac action potential. The most common genetic arrhythmic syndromes are caused by mutations in the KCNQ1 gene, the gene encoding K_V_7.1, underlying 30–35% of all LQTS subtypes (Figure 3). Any loss-of-function or gain-of-function mutation in this gene can induce severe arrhythmic phenotypes such as LQT1, Romano–Ward, Lange–Nielsen, or short QT syndromes (SQTS). In addition, mutations in the KCNE1 gene, which encodes minK, can cause the same arrhythmic syndromes [22]. The channel is the result of the assembly of four subunits with architecture similar to that of each repeat found in Na_V_ or Ca_V_ channels. CaM interacts with two non-continuous segments, named helix A and helix B, located after the last transmembrane segment S6 [84,85,86] (Figure 2). Helix A contains an IQ site that, like in Na_V_ and Ca_V_ channels, interacts with the CaM C-lobe under low Ca^2+^ resting levels. Because K_V_7.1 channels are tetramers, four CaM molecules interact simultaneously with the channel, forming a CaM ring just underneath the membrane [87]. Mutations causing LQT1 that interfere with binding to CaM, disrupt channel trafficking, assembly, gating or both [85,86]. Most mutations localize into helix B (M520R, K526E, P535T, and R539W [85,88,89]), and other known pathological mutation map in the IQ motif (i.e., W392R [86], Table 1; Figure 3). There is general agreement that the N-lobe anchors CaM to helix B, maintaining EF1 and EF2 hands loaded with Ca^2+^ all the time under physiological conditions (this represents a remarkable example of a change in Ca^2+^ binding affinity upon target engagement: the affinity for Ca^2+^ is larger for the N-lobe than for the C-lobe when CaM clamps K_V_7 channels), and the C-lobe transmits Ca^2+^ signals to the pore [15,16,90,91].

The lipid molecule phosphatidylinositol 4,5-bisphosphate (PIP_2_) is essential for K_V_7.1 function, and plays a critical role in coupling the voltage sensor to the gate of the pore. The auxiliary subunit KCNE1 causes a large (100 fold) increase in apparent affinity for PIP_2_, which leads to augmentation in current amplitude [95]. There are solid evidences for the formation of a ternary complex between PIP_2_, CaM, and K_V_7.1 channels. Interestingly, disruption of this complex by the LQT1 mutation K526E leads to compromised I_Ks_ channel gating [89].

At the bench, the role of Ca^2+^ binding to each EF-hand is often addressed by testing mutants in which the first aspartate on the Ca^2+^ binding loop is mutated to alanine. Depending on the hands affected, those mutants are named CaM1, CaM2. It has been found that overexpression of CaM3 or CaM4 affects the voltage-dependency of K_V_7.1 currents, causing a rightward shift in the current–voltage relationship (stronger depolarizations are required to open the channel), whereas Ca^2+^ leads to a displacement to the left (i.e. facilitates opening in response to depolarization) [16,90], suggesting that Ca^2+^-loading of the C-lobe is somehow transmitted to the voltage sensor of the channel. Interestingly, a cryo-EM structure reveals a potential interaction between the Ca^2+^-binding loop of the EF3 hand of the C-lobe and the S2-S3 loop which is part of the voltage sensor [87]. Consistent with the effect of CaM3 and CaM4 mutants, the pathological N98S mutant, that also disrupts Ca^2+^ binding to EF3, causes a shift in the K_V_7.1 voltage dependence of activation in the positive direction [87]. Given the similar impact on voltage-dependence caused by CaM3 and CaM4, it can be anticipated that those pathological mutations that disrupt Ca^2+^ binding to EF4 hand will also cause a shift on the voltage-dependence of K_V_7 channels. Furthermore, these results suggest an intimate connection between EF3 and EF4 hands, or that the K_V_7.1_AB/CaM complex may reorient to allow the EF4 hand to directly interact with the S2-S3 loop.

Recently, a mutation in the IQ site of the CaMBD of K_V_7.1 channels has been linked to gingival fibromatosis (GF), a disease related to human growth [92] (Table 2; Figure 3). This mutation (P369L), that does not compromise appreciably the integrity of the K_V_7.1_AB/CaM complex, leads to increased current levels, and is associated with reduced pituitary hormone secretion from AtT-20 cells. Another mutation linked to GF, R116L, is located in the N-terminal region, outside the CaMBD, and also causes an increase in current levels [92]. Interestingly, the N-terminal region of K_V_7.2 channels is the site of engagement of protein phosphatase 1, which regulates the phoshorylation state of resident CaM. Mutations at the N-terminus that disrupt binding of K_V_7.2 to this phosphatase cause an increase in current density [96,97]. However, it is not known if protein phosphatase 1 or another phosphatase binds to sites at the N-terminal domain of K_V_7.1 channels. Of note, no major cardiac effects have been described for these GF patients [92].

## 8. Neuronal channels

Besides the cardiac consequences of mutations in CALM genes described above, neurological disturbances have been also noted [26]. Although possible brain injury secondary to cardiac arrest during early life has been pointed out as the most probable cause [26], the neurodevelopmental and excitability alterations resemble those prompted by mutations at the CaMBD of neuronal channels (see below). Indeed, neuronal K_V_7 channels and neuronal Na_V_ channels have a perfectly recognizable and functional IQ binding motif, and neurological disorders linked to mutations on this site have been described for Na_V_1.2 and K_V_7.2 channels, including epileptic syndromes, characterized by seizures as a result of excessive and abnormal neuronal activity (Table 3 and Table 4; Figure 3). The mutation R1918H in Na_V_1.2 associated to epilepsy [98] may disrupt the interaction with the linker between the C- and N-lobes observed in some crystal structures [99]. The H1853R and R1918H mutations provoke an increase of the persistent current, and CaM overexpression reduces that current to normal values, suggesting that these mutations cause a decrease in CaM binding affinity [66] (Table 3; Figure 3).

The neuronal homologs of K_V_7.1 channels, K_V_7.2 and K_V_7.3, combine to form M-channels, which are critical for the control of excitability. These subunits were discovered searching for the genetic basis of benign familial neonatal seizures (BFNS), a rare autosomal-dominant idiopathic epilepsy of the newborn [100,101,102]. Later, it was found that mutations on the KCNQ2 gene, which encodes K_V_7.2 subunits, also leads to more severe encephalopathic epilepsy, with poor prognosis (Table 4; Figure 3). This disease is now named KCNQ2-encephalopathy or IEE7. EE is a severe brain disorder of early age that manifest with seizures, cognitive, behavioral, and neurological deficits and sometimes early death [103,104].

As for K_V_7.1 channels, CaM binding to helix A and helix B is critical for exiting the endoplasmic reticulum and affects assembly. In addition, mutations that alter CaM binding disrupt targeting of the channels to the axon initial segment [111,117,118]. Contrary to I_Ks_, Ca^2+^ inhibits the M-current [119,120]. It is thought that, like for Ca_V_, Na_V_, and SK2 channels, the transduction mechanism involves two steps. The first step for these channels is anchoring the C-lobe to the IQ motif or its structural equivalent under resting Ca^2+^ conditions, thereby increasing the effective local concentration of the N-lobe near its target sequence. The second step is the engagement of the N-lobe with its corresponding binding site. After completion of the second step that requires Ca^2+^, Ca_V_ channels inactivate or SK channels open the pore [17,67]. A similar two-step process is at work for K_V_7.2 channels, but the initial step takes place between the N-lobe and helix B. Formation of the N-lobe/helix B complex facilitates binding of the C-lobe to helix A located in the same or in another subunit [14,116,121,122]. The C-lobe transmits a gating signal when occupied with Ca^2+^, which either opens cardiac K_V_7.1 channels, or closes neuronal K_V_7 channels [16,90,91].

Some EE-causing mutations are located at the N-lobe/helix B interface. One particular helix B mutation, M518V (M546V in isoform 1), leads to cellular degeneration, but it is unclear how this phenotype relates to CaM binding [106]. There are contrasting reports regarding the effect of the K526N (K554N in isoform 1) mutation on CaM binding, voltage-dependence, and membrane surface expression (Table 4; Figure 3). There is general agreement on an impact on PIP_2_ sensitivity and a tendency to increase current density [105,106,113]. Similar to other mutations at or close to the CaM binding surface, channels carrying this mutation are less efficient at controlling neuronal excitability [106].

Mutations at the helix A/C-lobe interface include L339R, W344R, L351F, L351V, and R353G (Table 4; Figure 3). All these mutations affect CaM binding, surface expression, and current density, with a reasonable degree of correspondence between these parameters [108,109,110,123]. Mutations at the R333 site, located at the periphery of the binding surface, differentially affect the interaction with CaM. Whereas the R333W mutant interferes with CaM binding, the R333Q mutation does not. However, both mutations affect channel trafficking [105,106] (Table 4; Figure 3).

Interestingly, there are pathological mutations located in a site denominated TW helix [124], which is downstream of the IQ motif (Table 4). Although those mutants (L356V, T359R, and Y362C) do not participate in the interacting surface with CaM, there is a clear impact on CaM binding that does not translate in changes in current density. Remarkably, the integrity of the TW site appears to be essential for CaM binding and function when the interacting surface at either helix A or helix B is perturbed [124]. CaM binding also affects the assembly of the C-terminal domain, and, in turn, helix D tetramerization influences CaM binding [116]. The pathological mutation L609R (L637R in isoform 1) in helix D disrupts tetrameric coiled-coil formation, upsets CaM-mediated potentiation, and makes the channels more resistant to PIP_2_ depletion [97,115].

## 9. TrpV4 Channels

Transient receptor potential vanilloid subtype 4 (TrpV4) channels are cation-nonselective Ca^2+^-permeable with a 6-TM membrane topology similar to that of voltage-dependent K^+^ channels, presenting a tetrameric arrangement as well. TrpV4 are polymodal receptors activated by a wide variety of chemical and physical stimuli that include membrane stretch, mild heat, endogenous metabolites and synthetic chemicals. CaM binds to a distal site located in the C-terminal region [125]. There is a site upstream of the CaMBD which has been proposed to maintain the channel in a non-conducting state, whereas activated CaM sterically interferes with this presumed auto-inhibitory domain. In this way, Ca^2+^-CaM could potentiate channel function in response to different stimuli.

Gain-of-function mutations in the auto-inhibitory site (E797K and P799L) turn the activity of TrpV4 channels mostly independent of Ca^2+^-CaM regulation [126] and cause skeletal dysplasia (SD; Table 5), characterized by abnormalities of cartilage and bone growth, resulting in abnormal shape and size of the skeleton and disproportion of the long bones, spine, and head.

## 10. SK2 Channels

A recent report points to apamin sensitive Ca^2+^-CaM-activated K^+^ channels (SK) as a possible co-factor for the action of pathological CaM variants [127]. The CaM apo-C-lobe binds to a motif conserved among the SK channel family (LRxxWL) that is structurally equivalent to the IQ motif [10]. The C-lobe remains Ca^2+^ free in some SK variants, whereas the N-lobe senses Ca^2+^ and gates the pore. As in voltage-dependent channels, the S4–S5 linker plays a critical role in gating, but, instead of a voltage sensor, is the N-lobe with the EF-hands opened due to Ca^2+^ occupancy which transmit the mechanical force to the S4-S5 linker of an adjacent subunit to gate the pore [17]. CALM mutations (N54I, F90L, N98S, D96V, and D130G) all reduce SK2 current density. Because the C-lobe remains indifferent to Ca^2+^ when bound to SK2 channels, it is remarkable that the Ca^2+^ sensitivity of SK channels increases significantly when engaged to the C-lobe D96V mutant. Of note, at a 1:3 ratio, closer to that imposed by genetic balance of one mutated allele and five wild-type, no major effect was observed for the D96V mutant, suggesting that this mutant lacks a dominant-negative effect on SK channel function [127].

In summary, mutations in CALM genes affect a plethora of targets, and it is expected that each contribute to some degree to the observed phenotype. In general, disruption of CaM interaction with its partners have multiple and variable consequences. Overall, as pointed out by Chung and collaborators, the multiple effects caused by direct or indirect disruption of the CaM binding interface have a synergistic influence, and, in conjunction exert more severe impacts on excitability and health [106].

## Figures and Tables

**Table 1:** Mutations in Na_V_1.5 channels influencing CaM binding. LQT3: long QT syndrome type 3; BrS: Brugada syndrome; IFM motif: inactivation motif; A D_III_–D_IV_ linker: A CaM binding domain within the D_III_–D_IV_ linker; B D_III_–D_IV_ linker: B CaM binding B domain within the D_III_–D_IV_ linker.

Na_V_1.5 Mutation	Location	Interacting Site	Disease	Reference
F1486L	IFM motif	/	LQT3	[73]
M1487L	IFM motif	/	LQT3	[74]
T1488R	A D_III_–D_IV_ linker	N-lobe	LQT3	[74]
E1489D	A D_III_–D_IV_ linker	N-lobe	LQT3	[74]
K1493R	A D_III_–D_IV_ linker	N-lobe	LQT3	[74]
Y1494N	A D_III_–D_IV_ linker	N-lobe	BrS	[72]
Y1495S	A D_III_–D_IV_ linker	N-lobe	LQT3	[73]
M1498T	A D_III_–D_IV_ linker	N-lobe	LQT3	[73]
L1501V	A D_III_–D_IV_ linker	/	LQT3/BrS	[72]
G1502S	A D_III_–D_IV_ linker	/	BrS	[73]
K1505N	D_III_–D_IV_ linker	/	LQT3/BrS	[74]
R1512W	D_III_–D_IV_ linker	/	BrS	[75]
I1521K	B D_III_–D_IV_ linker	EF hand/C-lobe	BrS	[72]
V1525M	B D_III_–D_IV_ linker	EF hand/C-lobe	BrS	[72]
K1527R	D_III_–D_IV_ linker	/	BrS	[76]
V1777M	Pre-EF hands	/	LQT3	[73]
T1779M	Pre-EF hands	/	LQT3/BrS	[73]
E1784K	EF hands	B D_III_–D_IV_ linker	LQT3	[73]
L1786Q	EF hands	B D_III_–D_IV_ linker	LQT3/BrS	[73]
S1787N	EF hands	B D_III_–D_IV_ linker	LQT3	[73]
D1790G	EF hands	B D_III_–D_IV_ linker	LQT3/BrS	[66]
Y1795InsD	EF hands	B D_III_–D_IV_ linker	LQT3/BrS	[77]
Y1795C	EF hands	B D_III_–D_IV_ linker	LQT3	[66]
D1819N	EF hands	B D_III_–D_IV_ linker	LQT3	[73]
L1825P	EF hands	B D_III_–D_IV_ linker	LQT3	[77]
R1826H	EF hands	B D_III_–D_IV_ linker	LQT3	[77]
Q1832E	EF hands	B D_III_–D_IV_ linker	BrS	[77]
D1839G	EF hands	B D_III_–D_IV_ linker	LQT3	[73]
H1849R	EF hands	B D_III_–D_IV_ linker	LQT3	[73]
C1850S	EF hands	B D_III_–D_IV_ linker	BrS	[73]
V1861I	EF hands	B D_III_–D_IV_ linker	BrS	[73]
K1872N	EF hands	B D_III_–D_IV_ linker	BrS	[73]
M1875T	Pre-IQ motif	/	LQT3	[73]
R1897W	Pre-IQ motif	/	LQT3	[78]
E1901Q	Pre-IQ motif	/	LQT3	[66]
S1904L	Pre-IQ motif	/	LQT3/BrS	[74]
Q1909R	IQ motif	C-lobe	LQT3	[66]
R1913H	IQ motif	C-lobe	LQT3	[66]
A1924T	Post-IQ motif	/	BrS	[71]
G1935S	Post IQ motif	/	BrS	[74]

**Table 2:** Mutations in K_V_7.1 channels influencing CaM binding. GF: gingival fibromatosis; LQT1: long QT syndrome type 1; hA: helix A; hTW: TW helix; hB: helix B.

K_V_7.1 Mutation	Location	Interacting Site	Disease	Reference
P369L	hA	C-lobe	GF	[92]
A371T	hA	C-lobe	LQT1	[73]
A372D	hA	C-lobe	LQT1	[73]
S373P	hA	C-lobe	LQT1	[73]
L374H	hA	C-lobe	LQT1	[73]
W379S	hA	C-lobe	LQT1	[73]
R380S	hA	C-lobe	LQT1	[73]
S389Y	hTW	/	LQT1	[88]
T391I	hTW	/	LQT1	[73]
W392R	hTW	/	LQT1	[73]
K393M	hTW	/	LQT1	[73]
K393N	hTW	/	LQT1	[73]
R397N	hTW	/	LQT1	[73]
P448R	hTW-hB linker	/	LQT1	[73]
R451Q	hTW-hB linker	/	LQT1	[73]
R452W	hTW-hB linker	/	LQT1	[73]
G460S	hTW-hB linker	/	LQT1	[73]
I517T	hB	N-lobe	LQT1	[73]
R518G	hB	N-lobe	LQT1	[73]
R518P	hB	N-lobe	LQT1	[73]
R519C	hB	N-lobe	LQT1	[73]
M520R	hB	N-lobe	LQT1	[73]
V524G	hB	N-lobe	LQT1	[73]
A525T	hB	N-lobe	LQT1	[93]
K526E	hB	N-lobe	LQT1	[89]
R533W	Post-hB	/	LQT1	[94]
P535T	Post-hB	/	LQT1	[73]
R539W	Post-hB	/	LQT1	[85]

**Table 3:** Mutations in Na_V_1.2 channels influencing CaM binding. OS: Ohtahara syndrome; IGE: idiopathic generalized epilepsy.

Na_V_1.2 Mutation	Location	Interacting Site	Disease	Reference
H1853R	Pre-IQ motif	/	OS	[66]
R1918H	IQ motif	C-lobe	IGE	[66]

**Table 4:** Mutations in K_V_7.2 channels influencing CaM binding. EE: epileptic encephalopathy; BFNS: benign familial neonatal seizures; hA: helix A; hTW: TW helix; hB: helix B; hD: helix D.

K_V_7.2 Mutation		Location	Interacting Site	Location	Reference
Isoform 1	Isoform 3				
R333Q	R333Q	Pre-hA	/	EE	[105]
R333W	R333W	Pre-hA	/	EE	[106]
A337G	A337G	hA	C-lobe	EE	[107]
L339R	L339R	hA	C-lobe	BFNS	[108,109,110,111]
W344R	W344R	hA	C-lobe	BFNS	[109]
L351F	L351F	hA-hTW linker	/	BFNS	[109]
L351V	L351V	hA-hTW linker	/	BFNS	[109]
R353G	R353G	hA-hTW linker	/	BFNS	[108,109,110,111]
L356V	L356V	hTW	/	BFNS	[107]
T359K	T359K	hTW	/	EE	[112]
Y362C	Y362C	hTW	/	BFNS	[109]
M546V	M518V	hB	N-lobe	EE	[106,109]
R547W	R519W	hB	N-lobe	EE	[107]
K552T	K524T	hB	N-lobe	EE	[107]
R553Q	R525Q	hB	N-lobe	EE	[107]
K554N	K526N	hB	N-lobe	EE	[105,106,113]
K556E	K528E	hB	N-lobe	EE	[107]
R560W	R532W	hB	N-lobe	EE	[106]
P561L	P533L	hB	N-lobe	EE	[107]
D563N	D535N	hB	N-lobe	EE	[107]
R581Q	R553Q	hB	N-lobe	EE	[106,109]
R581G	R553G	hB	N-lobe	EE	[114]
L637R	L609R	hD	N-lobe	BFNS	[115,116]

**Table 5:** Mutations in TrpV4 channels influencing CaM binding. SD: skeletal dysplasia.

TrpV4 Mutation	Location	Interacting Site	Disease	Reference
E797K	Pre-CaMBD	unknown	SD	[126]
P799L	Pre-CaMBD	unknown	SD	[126]

## Abbreviations

BFNS	Benign familial neonatal seizures
BrS	Brugada syndrome
CaM	Calmodulin
CaMBD	CaM-binding domains
Ca_V_	Voltage-gated Ca^2+^-channels
CDI	Ca^2+^-dependent inactivation
CDR	Ca^2+^-dependent regulator
CPVT	Catecholaminergic polymorphic ventricular tachycardia
D_I_–D_IV_	Homologous repeat domains
ECC	Excitation–contraction coupling
EE	Epileptic encephalopathy
GF	Gingival fibromatosis
hA	Helix A
hB	Helix B
hC	Helix C
hD	Helix D
hiPSC-CM	Human induced pluripotent stem cell-derived cardiomyocytes
hTW	TW helix
IGE	Idiopathic generalized epilepsy
I_Ks_	Slow delayed rectifier potassium current
IVF	Idiopathic ventricular fibrillation
K_V_7	Voltage-gated K^+^ channels 7
LQTS	Long QT syndrome
Na_V_	Voltage-gated Na^+^-channels
NCX	Na^+^-Ca^2+^ exchanger
NSCaTE	N-terminal spatial Ca^2+^ transforming element
OS	Ohtahara syndrome
PIP_2_	Phosphatidylinositol 4,5-bisphosphate
RyR	Ryanodine receptor ion channels
S1–S6	Six transmembrane segments
SCD	Sudden cardiac death
SD	Skeletal dysplasia
SK	Small conductance calcium activated potassium channel
SQTS	Short QT syndromes
SR	Sarcoplasmic reticulum
TrpV4	Transient receptor potential vanilloid subtype 4
